# Supplementary material for: Causal Relationship between Aging and Anorexia Nervosa: A White-Matter-Microstructure-Mediated Mendelian Randomization Analysis
Source: Biomedicines. 2024 Aug 16;12(8):1874. doi: 10.3390/biomedicines12081874 (PMC11351342; doi:10.3390/biomedicines12081874)
Supplement: Supplementary file 1 [file biomedicines-12-01874-s001.zip › Supplementary Figure/Figure S9/Leaveoneout plot of MR analysis for ubm-b-1964 to AN.pdf]

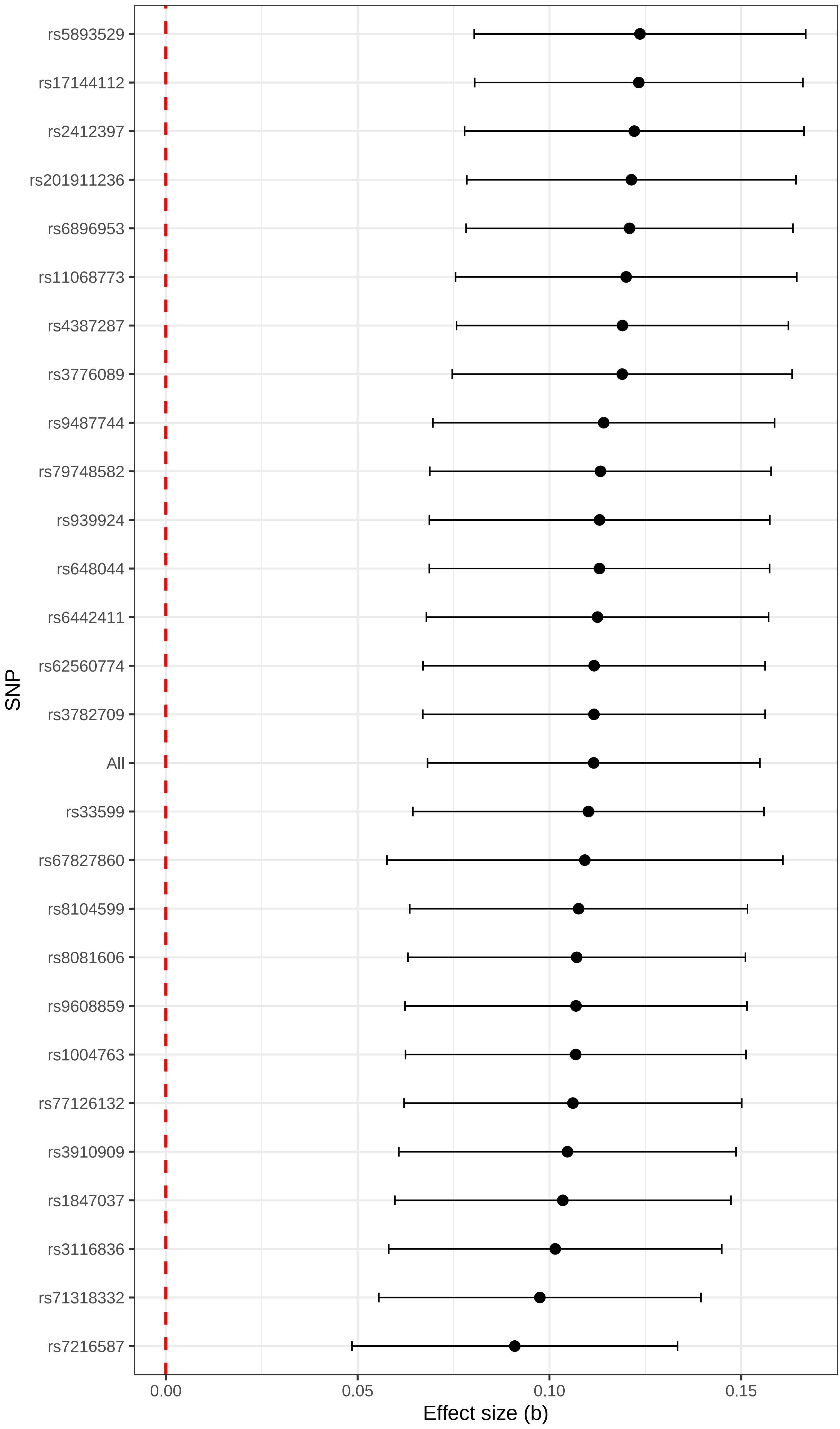

rs5893529
rs17144112
rs2412397
rs201911236
rs6896953
rs11068773
rs4387287
rs3776089
rs9487744
rs79748582
rs939924
rs648044
rs6442411
rs62560774
rs3782709
All
rs33599
rs67827860
rs8104599
rs8081606
rs9608859
rs1004763
rs77126132
rs3910909
rs1847037
rs3116836
rs71318332
rs7216587
SNP
0.00
0.05
0.10
0.15
Effect size (b)
